# Premature Acute Myocardial Infarction in a Competitive Athlete: Exploring the Potential Impact of Omega-3/Omega-6 Ratio

**DOI:** 10.1016/j.jaccas.2026.109226

**Published:** 2026-07-15

**Authors:** Bragato Maria Chiara, Pina Alessandra, Leidi Filippo, Ivan Calchera, Tresoldi Simone, Pozzi Matteo, Lettino Maddalena, Vandoni Pietro, Angela Maria Rizzo, Righetti Stefano

**Affiliations:** a Interventional Cardiology, Fondazione IRCCS San Gerardo dei Tintori, Monza, Italy; b School of Medicine and Surgery, University of Milano-Bicocca, Milan, Italy; c University of Adelaide, Adelaide, South Australia, Australia; d Intensive Care Unit, Fondazione IRCCS San Gerardo dei Tintori, Monza, Italy; e Cardiothoracic and Vascular Department, Fondazione IRCCS San Gerardo dei Tintori, Monza, Italy; f Department of Pharmacological and Biomolecular Sciences, University of Milan, Milan, Italy

**Keywords:** cardiac assist device, exercise, hemodynamics, myocardial infarction, percutaneous coronary intervention, risk factor

## Abstract

**Background:**

Acute myocardial infarction is increasing in younger adults. Epidemiologic studies suggest a low Omega-3/Omega-6 ratio may contribute to cardiac events. Athletes on a Western diet and high training loads have reduced Omega-3/Omega-6 ratios.

**Case Summary:**

A 29-year-old man with an unremarkable medical history experienced cardiac arrest during a soccer match. Coronary angiography revealed an atherosclerotic plaque occluding the proximal left anterior descending artery, treated with 2 drug-eluting stents. The patient was admitted to intensive care; brain death was declared the next day. No cardiovascular risk factors were identified, except for a reduced Omega-3/Omega-6 ratio.

**Discussion:**

A young athlete without classical risk factors but with a low Omega-3/Omega-6 ratio developed unstable coronary disease. This association warrants further investigation.

**Take-Home Messages:**

This case highlights the potential role of Omega-3/Omega-6 ratios in cardiovascular risk in young athletes and underscores the importance of performing immediate cardiopulmonary resuscitation before advanced medical services arrive.

## Visual Summary

**Figure:**
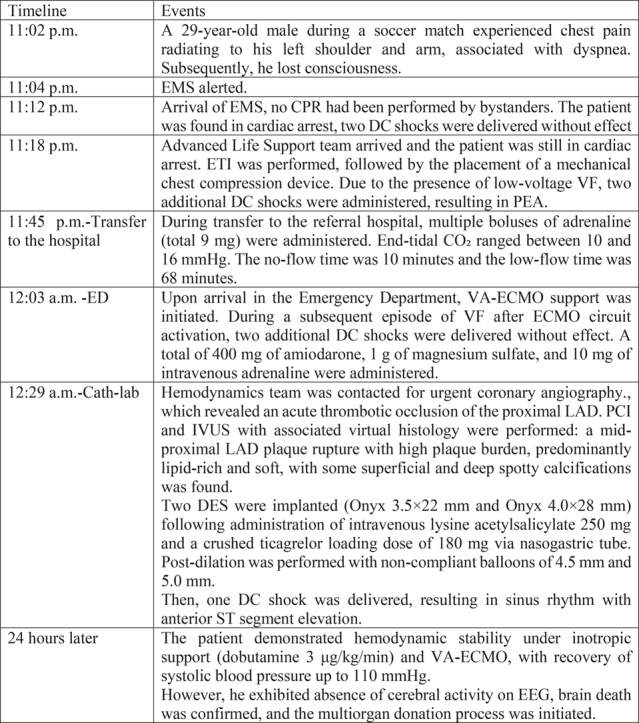

## History of Presentation

A 29-year-old man experienced chest pain during a soccer match radiating to the left shoulder and arm, associated with dyspnea. Subsequently, he developed cardiac arrest. No bystander cardiopulmonary resuscitation (CPR) was performed. Emergency medical service was alerted immediately. The first medical contact occurred 10 minutes later, and the patient was found in cardiac arrest.

## Past Medical History

The patient had an unremarkable past medical history, no cardiovascular risk factors, and no family history of cardiac disease or acute myocardial infarction. He was neither a smoker nor a user of vaping products. He was completely asymptomatic until the event. Three months before, he underwent an electrocardiogram at rest and during stress testing, which showed sinus rhythm with bradycardia and no significant exercise-induced abnormalities.

## Differential Diagnosis

The differential diagnosis included acute myocardial infarction due to coronary occlusion, coronary dissection, congenital coronary artery anomalies, aortic dissection, and cardiomyopathies.

## Investigations

Upon emergency medical service arrival, the rhythm was ventricular fibrillation (VF).

## Management

Two direct current (DC) shocks were delivered without effect. The Advanced Life Support team arrived 6 minutes later. The patient was still in cardiac arrest, and endotracheal intubation was performed, followed by the placement of a mechanical chest compression device. Because of low-voltage VF, 2 additional DC shocks were administered, resulting in pulseless electrical activity. During transfer to the referral hospital, multiple boluses of adrenaline (total 9 mg) were administered. End-tidal CO_2_ ranged between 10 and 16 mm Hg. Upon arrival in the Emergency Department, the patient was still in pulseless electrical activity. Echocardiography showed absence of left ventricular mechanical activity, a nondilated aorta, a normal right ventricle, and no pericardial effusion. Given the refractory cardiac arrest, veno-arterial extracorporeal membrane oxygenation (VA-ECMO) was initiated. The patient remained in VF after VA-ECMO initiation despite an additional DC shock and further amiodarone administration. The no-flow time was 10 minutes, and the low-flow time was 68 minutes. Given the clinical presentation, the patient was referred for urgent coronary angiography. Coronary angiography revealed an acute thrombotic occlusion of the proximal left anterior descending (LAD) artery (Figure 1, Video 1). Percutaneous coronary intervention was performed: After placement of coronary guidewires in the distal LAD artery and diagonal branch, thromboaspiration was attempted but failed to restore adequate flow. Predilation with a 2.0-mm semicompliant balloon was then performed, achieving a distal thrombolysis in myocardial infarction flow of 1.

**Figure 1:**
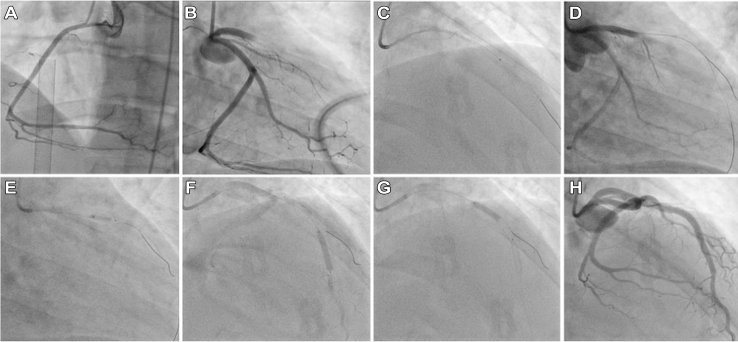
Coronary Angiography and Percutaneous Coronary Intervention (A) Right coronary angiography; (B) proximal left anterior descending artery occlusion; (C and D) ineffective thrombus aspiration; (E) predilation with a 2.0-mm semicompliant balloon; (F and G) implantation of 2 drug-eluting stents in the mid-proximal left anterior descending artery; (H) optimal final angiographic result.

Intravascular ultrasound with associated virtual histology (Figure 2) demonstrated a mid-proximal LAD artery plaque rupture with high plaque burden, predominantly lipid-rich and soft, with some superficial and deep spotty calcifications. Two drug-eluting stents (3.5 × 22 mm and 4.0 × 28 mm) were implanted following administration of intravenous lysine acetylsalicylate 250 mg and a crushed ticagrelor loading dose of 180 mg via a nasogastric tube. Postdilation was performed with noncompliant balloons (4.5 and 5.0 mm), and normal flow was achieved (Video 2). After coronary revascularization, 1 DC shock was delivered, resulting in return to sinus rhythm, with diffuse anterior ST-segment elevation and Q waves in V_1_ to V_5_ (Figure 3).

**Figure 2:**
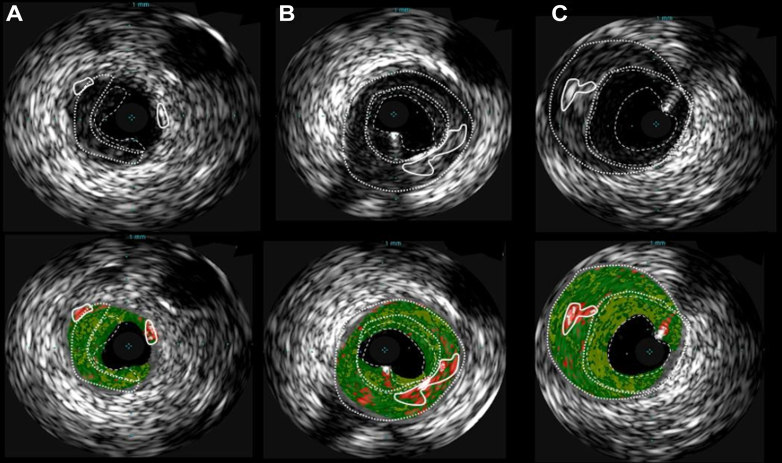
Plaque Assessment With Intravascular Ultrasound and Virtual Histology (A) Plaque in the mid-left anterior descending artery; (B) plaque in the mid-proximal left anterior descending artery, just upstream of the bifurcation with the first diagonal branch; (C) plaque in the proximal left anterior descending artery. Dashed line: thrombotic component; dotted line: fibro-lipidic component; solid line: intraplaque calcifications.

**Figure 3:**
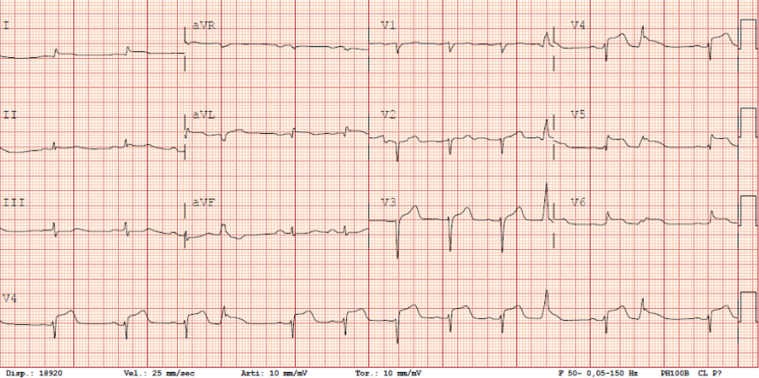
Electrocardiogram After Return of Spontaneous Circulation After coronary revascularization and direct current shock, the electrocardiogram showed sinus rhythm, with diffuse anterior ST-segment elevation and Q waves in V_1_ to V_6_. I, II, III, aVF, aVL, aVR, V_1_ to V_6_ = electrocardiographic leads.

At the end of the procedure, mean arterial pressure with VA-ECMO support was 55 mm Hg, heart rate was 88 beats/min, and echocardiography demonstrated a left ventricular ejection fraction of 15% to 20% with diffuse hypokinesia and akinesia of the apex and the entire anterior wall.

## Outcome and Follow-Up

At 24 hours after the event, the patient demonstrated clinical stability under inotropic support (dobutamine 3 μg/kg/min) and VA-ECMO, with recovery of systolic blood pressure up to 110 mm Hg. Echocardiography showed progressive improvement in left ventricular ejection fraction to 40% to 45%, with persistent apical and mid-anterior wall akinesia. However, the patient exhibited clinical criteria for brain death determination, which was confirmed according to local regulations. Laboratory tests revealed normal cardiovascular risk factors: glycated hemoglobin 33 mmol/mol, triglycerides 157 mg/dL, total cholesterol 151 mg/dL, high-density lipoprotein 28 mg/dL, low-density lipoprotein (LDL) 91 mg/dL. Blood and urine toxicology screening was negative for substances of abuse and anabolic steroids. Neither lipoprotein(a) measurement nor genetic testing was performed at presentation. Lipidomic analysis of red blood cell membranes showed a reduced Omega-3 Index (4.6%) and a markedly low plasma eicosapentaenoic acid-to-arachidonic acid (EPA/AA) ratio (0.10).

## Discussion

We described a rare case of premature acute myocardial infarction due to occlusive coronary thrombosis on atherosclerotic plaque rupture in a 29-year-old patient. In certain individuals, for reasons that are still not fully understood, atherosclerotic plaques with features of advanced and chronic disease, including calcification, may develop at a very young age. It is established that occlusive coronary thrombosis in patients younger than 35 years is associated with risk factors such as smoking, elevated LDL-cholesterol, substance abuse, or a family history of ischemic heart disease. 1 Our patient had none of these conditions. Moreover, intravascular ultrasound revealed rupture of a fibro-lipidic coronary plaque with spotty calcifications, despite no known risk factors. The only abnormal finding was the Omega-3 and Omega-6 content in the blood. Although limited data prevent definitive classification of these indices as cardiovascular risk factors, protective Omega-3 Index values have been reported to exceed 8%. Omega-3 Index values between 4% and 8% are considered to carry “intermediate” cardiovascular risk, and values <4% carry the highest cardiovascular risk.^2^ Although our athlete did not have an Omega-3 Index below 4%, the value was very close to this threshold; moreover, he exhibited a markedly low plasma EPA/AA ratio, lower than that reported by Serikawa et al^3^ in patients younger than 50 years with acute coronary syndromes and lower than the values shown to be protective in the JELIS (Japan EPA Lipid Intervention Study) trial.^4^ The plasma EPA/AA ratio is associated with acute coronary syndromes in young individuals in multivariate analyses.

Athletes have low blood Omega-3 content.^5^ High training loads may reduce blood Omega-3 levels through mechanisms that are not yet understood.^5^ When combined with a diet low in Omega-3 and high in Omega-6 intake, typical of Western people,^6^ this may result in a low Omega-3/Omega-6 ratio in athletes, well below levels considered protective against cardiovascular disease.^5^ Moreover, demographic studies showed that individuals younger than 40 years tend to have higher Omega-6 levels, resulting in a lower Omega-3/Omega-6 ratio than older individuals, and thus potentially reduced protection against cardiac events.^7^ It is possible that other, as yet poorly understood, factors contributed to the event, beyond the patient’s blood Omega-3 concentration. A limitation of this report is that neither lipoprotein(a) measurement nor genetic testing was performed. Although elevated lipoprotein(a) levels and/or genetic factors may have contributed to disease development, the patient’s young age, normal LDL-cholesterol levels, and presence of calcified coronary plaques suggest a multifactorial pathophysiology, in which the markedly altered EPA/AA ratio may have acted as an additional contributor. Two potential associations warrant further dedicated studies: 1) the link between a low Omega-3/Omega-6 ratio in young individuals—more pronounced than in those older than 45 years^7^—and the stable or even rising incidence of premature myocardial infarction and 2) the relationship between reduced Omega-3 content in highly trained athletes and the extent of coronary artery disease in athletes with a low cardiovascular risk profile.^8^

Such events may be unpreventable despite adequate sports eligibility assessment, including an electrocardiogram and exercise testing. Three months before, screening showed a normal electrocardiogram and exercise test. Per current sports cardiology guidelines, there was no indication for further testing, such as coronary computed tomography.^9^

This highlights the importance of disseminating basic life support techniques and automated external defibrillator (AED) use among the public. Our patient experienced prolonged no-flow and low-flow intervals because of the absence of bystander intervention.

Post–cardiac arrest prognosis depends on the time to circulatory support initiation. Policies promoting prompt CPR and rapid AED deployment in athletic settings significantly improve survival outcomes.

The most critical determinant of survival is the interval from collapse to defibrillation, with survival rates declining by 7% to 10% for every minute of delay.^10^

This concept is emphasized both in the guidelines and in the state-of-the-art review on sudden cardiac death in athletes.^9^^,^^10^ Reported survival rates in athletic venues with prompt AED use and bystander CPR have reached as high as 67% to 75% at hospital discharge, and up to 89% among collegiate athletes, reflecting the impact of well-implemented emergency action plans and AED accessibility.^10^

## Conclusions

A 29-year-old soccer player experienced an acute myocardial infarction complicated by cardiac arrest, caused by rupture and thrombosis of an atherosclerotic coronary plaque with features of chronic disease. The patient had no classical cardiovascular risk factors; however, he had a low blood Omega-3/Omega-6 ratio. Currently, limited data prevent defining these indices as cardiovascular risk factors. Indeed, the association between athletes following a Western diet with high training loads and a reduced Omega-3/Omega-6 ratio warrants further investigation. A deeper investigation of this topic could help explain and potentially address the rising incidence of premature myocardial infarction and coronary artery disease in athletes with a low traditional cardiovascular risk profile.

Also, this case underscores the importance of the dissemination of basic life support-defibrillation techniques to immediately start CPR to minimize the delay of intervention preceding the arrival of advanced medical services.

## Funding Support and Author Disclosures

The authors have reported that they have no relationships relevant to the contents of this paper to disclose.

**Take-Home Messages**

• The Omega-3/Omega-6 ratio may play a potential role in shaping cardiovascular risk profiles in young athletes; therefore, a deeper investigation of this topic could lead to a better understanding of the etiology of myocardial infarction in young patients.
• Widespread dissemination of basic life support-defibrillation techniques in the general public is crucial for the survival of athletes experiencing an unexpected cardiac event.

## Appendix

Video 1 Baseline Coronary Angiography Thrombotic occlusion of the proximal left anterior descending artery.

Video 2 Coronary Angiography After Percutaneous Coronary Intervention Normal flow restored in the left anterior descending artery.

Video 3 Video Summary of the Case
